# Poly(3-Hydroxybutyrate)-Based Nanoparticles for Sorafenib and Doxorubicin Anticancer Drug Delivery

**DOI:** 10.3390/ijms21197312

**Published:** 2020-10-03

**Authors:** György Babos, Joanna Rydz, Michal Kawalec, Magdalena Klim, Andrea Fodor-Kardos, László Trif, Tivadar Feczkó

**Affiliations:** 1 Institute of Materials and Environmental Chemistry, Research Centre for Natural Sciences, Magyar tudósok körútja 2, H-1117 Budapest, Hungary; babos@mukki.richem.hu (G.B.); kardos@mukki.richem.hu (A.F.-K.); trif.laszlo@ttk.hu (L.T.); 2 Research Institute of Biomolecular and Chemical Engineering, Faculty of Engineering, University of Pannonia, Egyetem u. 10, H-8200 Veszprém, Hungary; 3 Centre of Polymer and Carbon Materials Polish Academy of Sciences, 34, M. Curie-Skłodowskiej Str., 41-819 Zabrze, Poland; jrydz@cmpw-pan.edu.pl (J.R.); michal.kawalec@cmpw-pan.edu.pl (M.K.); mklim@cmpw-pan.edu.pl (M.K.); 4 Department of Microbiology and Virology School of Pharmacy with the Division of Laboratory Medicine Medical University of Silesia, 4 Jagiellońska St., 41-200 Sosnowiec, Poland

**Keywords:** poly(3-hydroxybutyrate), sorafenib, doxorubicin, dual drug-loaded nanoparticles

## Abstract

Dual drug-loaded nanotherapeutics can play an important role against the drug resistance and side effects of the single drugs. Doxorubicin and sorafenib were efficiently co-encapsulated by tailor-made poly([R,S]-3-hydroxybutyrate) (PHB) using an emulsion–solvent evaporation method. Subsequent poly(ethylene glycol) (PEG) conjugation onto nanoparticles was applied to make the nanocarriers stealth and to improve their drug release characteristics. Monodisperse PHB–sorafenib–doxorubicin nanoparticles had an average size of 199.3 nm, which was increased to 250.5 nm after PEGylation. The nanoparticle yield and encapsulation efficiencies of drugs decreased slightly in consequence of PEG conjugation. The drug release of the doxorubicin was beneficial, since it was liberated faster in a tumor-specific acidic environment than in blood plasma. The PEG attachment decelerated the release of both the doxorubicin and the sorafenib, however, the release of the latter drug remained still significantly faster with increased initial burst compared to doxorubicin. Nevertheless, the PEG–PHB copolymer showed more beneficial drug release kinetics in vitro in comparison with our recently developed PEGylated poly(lactic-co-glycolic acid) nanoparticles loaded with the same drugs.

## 1. Introduction

The treatment of primer liver tumors (hepatocellular carcinoma, HCC) is not sufficiently efficient because of the late recognition [1]. Chemotherapy alone or combined with surgery or radiotherapy are the most frequent protocols. Sorafenib is the unique Food and Drug Administration (FDA, US)-approved multikinase inhibitor against advanced HCC. The drug targets signaling pathways, inhibits kinase enzymes, blocks growth factors thereby suppresses tumor growth and restrains angiogenesis. Doxorubicin is a widely used anthracycline drug belonging to a DNA intercalator. It blocks topoisomerase II enzyme and thereby disrupts the DNA replication and tumor cell proliferation. However, in many cases the drug causes serious side effects. The chance of survival is significantly reduced, when the tumor cells develop resistance against the drug agent [2]. Hence, combination therapy is also used in the clinical practice. While the efficiency and also the cost effectiveness of the combined therapies have been increased, the decrease in side-effects is still not satisfactory [3].

Nanotherapeutics can overcome the drawbacks of traditional medicines, such as overdose and severe side effects. Some kinds of commercial drug delivery systems have already verified their efficacy, such as lyposomes (e.g., Doxyl) [4], modified viruses (Rexin G) [5], protein-bound nanoparticles (Abraxane) [6] or biodegradable polymer-based depots [7]. Polymeric nanoparticles may increase the water-solubility and circulation half-life of drugs, hence improving their bioavailability. They can also control the drug release and contain active targeting moieties besides passive enhanced permeability and retention effect. Currently, drug-loaded nanoparticles are usually prepared using polylactide [8], poly(lactic-*co*-glycolic acid) (PLGA) [9] and poly(-caprolactone) [10] or their copolymers especially with poly(ethylene glycol) (PEG) [11,12]. PEGylation of the particles is a promising method to increase the lifetime of the nanoparticles in the blood stream [13].

Poly(3-hydroxybutyrate) (PHB) has been originally investigated as a biodegradable packaging material [14,15,16]. PHB has high crystallinity, a relatively high melting point and sufficient hydrolytic stability. These features enable its use for biomedical purposes. [17] Its copolymers/composites can be utilized in bone implants [18,19], however, during the last decade PHB-based nanoparticles have been also considered as promising drug delivery systems. They are not only biodegradable but also bioresorbable which means that they can be eliminated through natural pathways by filtration or metabolism. [20] The degradation occurs by the hydrolysis of the ester bonds. PHB nanoparticles and low molecular weight polymer fragments can be phagocytosed by macrophages and degraded intracellularly. Its degradation product, 3-hydroxybutyric acid, is physiologically present in the human blood [21], hence its appropriate derivatives have no acute cytotoxicity. [22] PHBs can be synthesized both naturally (microbial) or synthetically. [23] The compounds have a broad molecular weight range. Smaller molecules with mass-average molecular weight (*M_w_*) a few hundred to thousand are liquid-like, and those in the hundreds of thousands range *M_w_* are solid, suitable for the manufacture of medical implants [24]. One major disadvantage of naturally produced isotactic PHB is its high degree of crystallinity. Natural as well as synthetic PHBs have thus been conjugated with various polymers such as PEG [21], poly(*ε*-caprolactone) [17], poly(2-(dimethylamino)ethyl methacrylate) [25] and chitosan [26]. Poly(3-hydroxybutyrate-co-3-hydroxyvalerate) is a copolymer of PHB with widely studied applications in implants, tissue engineering and the nanoparticulate delivery of drugs such as anticancer agents [27,28]. The drug delivery applications of PHB has been recently widened to antimicrobial [29,30] and cancer treatment [21], gene delivery [25] and topical drug delivery [31]. In addition to nanoparticles, other types of nanocomposites, for example conjugated with liposomes [31], have also been developed. Nanoparticles are commonly obtained by nanoprecipitation, emulsion techniques (single or double), or electrospraying methods [22]. Recently, we co-encapsulated sorafenib and doxorubicin using PLGA and PEGylated PLGAs [11].

However, the release kinetics of PHB nanoparticles has not yet been extensively characterized; and for other types of polymeric nanoparticles, generally three types of mechanisms have been described [32]: monophasic release with diffusion; biphasic release with a high initial burst followed by saturation; triphasic profile starting with an initial burst, then polymer degradation and ended with a slow diffusion release.

Drug delivery profiles of polymeric nanoparticles can be divided in three phases: the first one corresponding to the burst effect that is followed by a slower release due to drug diffusion through water-filled pores and through the polymeric matrix, and finally with a fast release occurring because of the polymer matrix erosion. [22] In our recent work, sorafenib and doxorubicin release from PLGA and PEGylated PLGA nanoparticles was investigated in which triphasic release profiles could be observed for both of the drugs.

In this work, atactic poly([*R,S*]-3-hydroxybutyrate) with a molecular weight 2200 g/mol was selected in preliminary tests as the most promising drug carrier, and it was optimized for sorafenib and doxorubicin co-encapsulation by emulsion–solvent evaporation method. PEGylated nanoparticles were also produced to extend their circulation half-life. Particle morphology, size, dispersity and yield as well as the encapsulation efficiency of the active agents were studied. The release kinetics of dual drug-loaded PHB and PEGylated PHB nanoparticles were also extensively studied.

## 2. Results and Discussion

### 2.1. Polymer Synthesis and Preliminary Nanoparticle Preparation

PHBs with different average molecular weights (Mw = 600 g/mol, 1300 g/mol, 1800 g/mol, 2200 g/mol) and PEGylated PHBs have been tested in nanoprecipitation and emulsion methods. The nanoprecipitation method resulted in doxorubicin and sorafenib co-loaded nanoparticles with poor encapsulation efficiency (mostly under detection limit) or of extreme dispersity (polydispersity index of polymer > 0.5). Polymers with a molecular weight ≤1800 g/mol were found to be highly soluble in water, hence low particle yields (<25%) were obtained, consequently Mw = 2200 was selected for further experiments.

### 2.2. Morphology and Size

**Figure 1:**
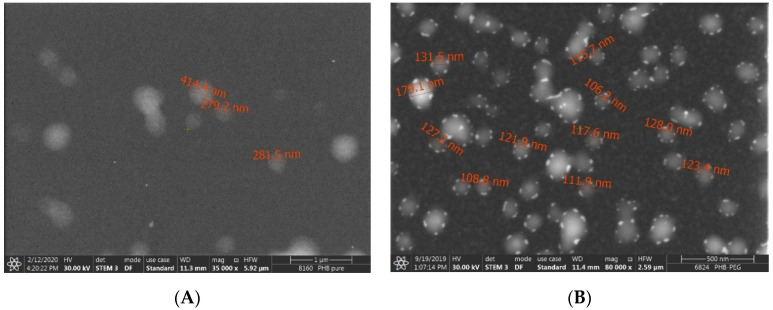
Scanning electronmicroscopic images of the PHB (**A**) and PEGylated PHB (**B**) nanoparticles.

Dual anticancer drug-loaded nanomedicines were formed by emulsion–solvent evaporation method. After the preparation process, PEG was conjugated to the surface of PHB–sorafenib–doxorubicin nanoparticles in order to prepare the stealth drug carrier from the PHB and to compare its features with the original nanopharmaceuticals. Both the non-PEGyylated and PEGylated (PEGylated PHB–sorafenib–doxorubicin) nanoparticles were found to be mostly spherical (Figure 1).

**Figure 2:**
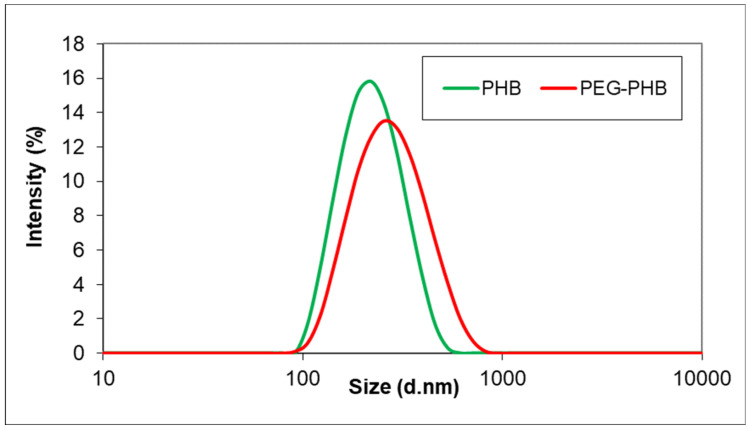
Size distribution of the PHB and PEGylated PHB nanoparticles.

The intensity mean average size of PHB–sorafenib–doxorubicin nanoparticles was 199.3 ± 6.5 nm with a monodisperse distribution (Figure 2) and low polydispersity index (0.071 ± 0.016). The size was increased by PEGylation, however, with the value of 250.5 ± 5.2 nm (polydispersity index: 0.155 ± 0.017) the PEGylated PHB–sorafenib–doxorubicin nanoparticles still remained in the desirable size range of polymeric nanoparticles, which is 50–300 nm, since the smaller ones can be rapidly excreted by the kidneys, while particles >300 nm are quickly recognized by the macrophages of the reticuloendothelial system [33].

### 2.3. Yield, Drug Loading and Encapsulation Efficiency

Important parameters such as drug loading and encapsulation efficiency (EE) indicate the quality of a drug delivery system. In this study, the PHB nanoparticles loaded with sorafenib and doxorubicin were produced by relatively high yield and encapsulation efficiencies for both drugs, which were decreased throughout the several steps of the PEGylation (Table 1). The doxorubicin loading was significantly lower related to that of sorafenib as a consequence of the lower weight of the former drug (0.5 mg vs. 1.5 mg sorafenib) added into the preparation process. The PEGylation did not influence the doxorubicin loading, although the sorafenib content of the nanoparticles was reduced probably because of the higher sorafenib content adsorbed on the surface of the nanomedicines as also experienced in the drug release experiments (see Section 3.5).

**Figure 3:**
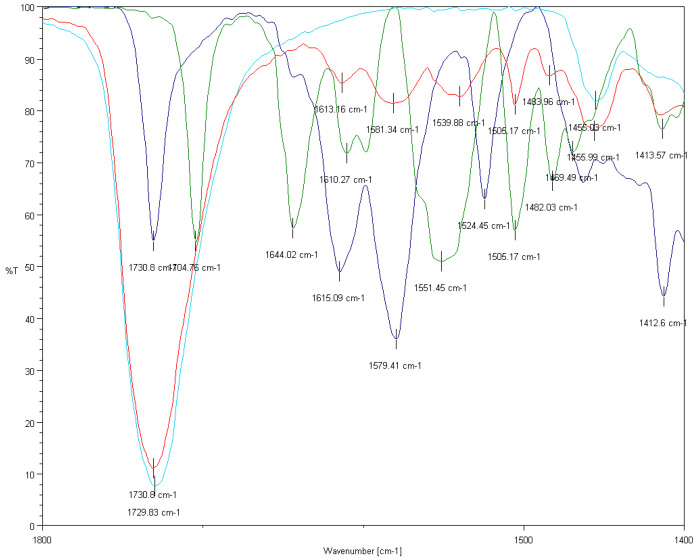
FTIR spectra of doxorubicin HCl salt (dark blue), sorafenib (green), PHB polymer (light blue) and dual drug-loaded PHB nanoparticles (red) in the 1400–1800 cm^−1^ wavenumber region.

The presence of the two anticancer components in the drug-loaded PHB nanoparticles can be identified in its spectra plotted in the 1400–1800 cm^−1^ region by Fourier transform infrared spectroscopy (Figure 3). Due to the fact that the fingerprint region of the two anticancer drugs as well as that of the PHB are absorption band-rich, the 1400–1800 cm^−1^ region proved to be suitable for the identification of the former components, since the polymer does not have strong absorption bands in the 1475–1650 cm^−1^ interval. The most unambiguous evidence of the presence of the doxorubicin in the PHB nanoparticles is the absorption band around 1580 cm^−1^ (1579 cm^−1^ in the spectra of doxorubicin HCl salt and 1581 cm^−1^ in the spectra of the PHB nanoparticles, which corresponds to the scissoring vibration of the amino group (neither the polymer nor the sorafenib have any absorption band in this region) [34,35]. The 1430–1625 cm^−1^ region is where the numerous aromatic stretching C=C vibrations are present, and the overlapping of these bands are also noticeable in the spectra of the composite sample. The presence of sorafenib is confirmed by the absorption band at 1505 cm^−1^, which corresponds to the disubstituted pyridine ring (stretching vibration of the C=N bond) [36].

**Figure 4:**
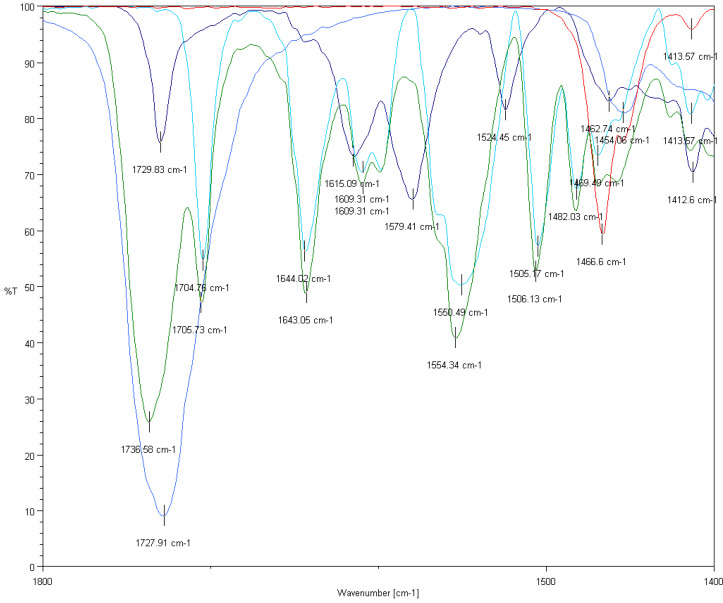
FTIR spectra of doxorubicin HCl salt (dark blue), sorafenib (light blue), PHB polymer (blue), amino-PEG (red) and dual drug-loaded PEGylated PHB nanoparticles (green) in the 1400–1800 wavenumber region.

While in the case of PHB nanoparticles the presence of both drugs was confirmed based on some specific absorption bands, in the PEGylated PHB nanoparticles the absorption bands specific to the sorafenib were dominating in the comparison (Figure 4). Many specific absorption bands of sorafenib (light blue) appear in the spectra of the drug containing the composite sample (green): 1705, 1643, 1609, 1554, 1506 and 1482 cm^−1^. However, there is no obvious evidence for the presence of doxorubicin in PEGylated PHB according to the FTIR results.

### 2.4. Thermal Analysis

**Figure 5:**
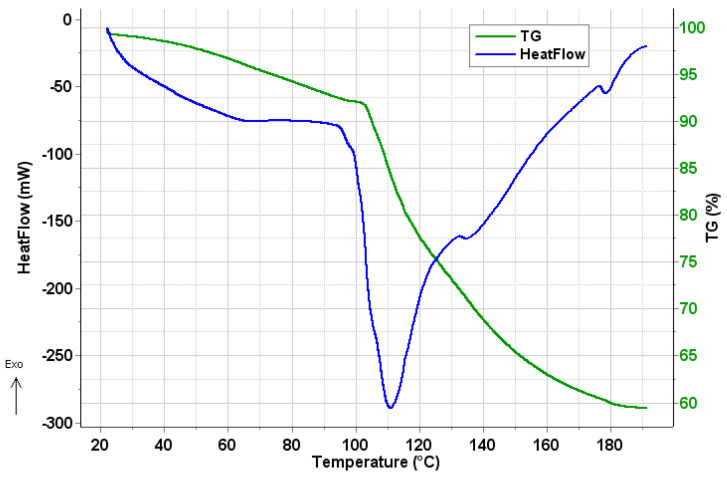
Mass (TG) and heat flow traces of the PHB polymer.

The presence of any residual DCM in the PHB polymer sample from the preparation process was investigated by thermogravimetry-differential scanning calorimetry coupled to mass spectrometric evolved gas analysis (TG–DSC–MSEGA) in the temperature interval of 20–190 °C. In Figure 5, the mass loss (TG) and calorimetric (heat flow) signals are plotted against temperature. It can be seen that from the starting of the measurement up to 98.7 °C, a mass loss of 7.9% occurs, which is accompanied by a small and broad endotherm. Above 98.7 °C up to the end of the measurement, another mass loss of 32.7% takes place. The much sharper, but also broad endotherm (extrapolated onset temperature: 100.2 °C; peak maximum temperature: 111.1 °C) with its two small satellite peaks at 135.5 °C and 178.4 °C corresponds to the evaporation of water, as confirmed by MSEGA.

**Figure 6:**
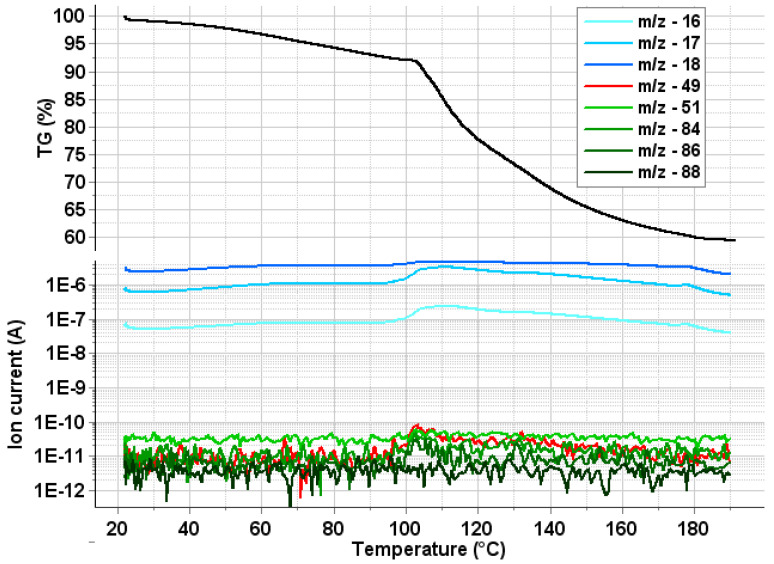
Mass (TG—upper part of the figure) and main characteristic ions of DCM (lower part of the figure).

The mass spectrometric evolving gas analysis results, together with the mass loss curve (TG) are shown in Figure 6. During the first mass loss step, weakly surface bound water desorbs, as proved by the slow rise of m/z 18, 17 and 16 ion current curves. Above 100 °C, the water captured inside the polymeric chains (more strongly bound) is released, as it can be seen as a much steeper rise of the curves characteristic to the water, and from the sharper endotherm accompanying with this mass loss step. According to the literature, the thermal degradation of the PHB starts at much higher temperatures (well above 200 °C) [37,38,39], so the water released in the second step cannot result from the thermal degradation of the polymer. During the measurements, no significant amount of DCM was detected by MSEGA in the evolved volatiles (the characteristic fragment ions of DCM, m/z 88, 86, 84, 51 and 49 are plotted in the lower part of the Figure 6).

### 2.5. In Vitro Drug Release Kinetics

**Figure 7:**
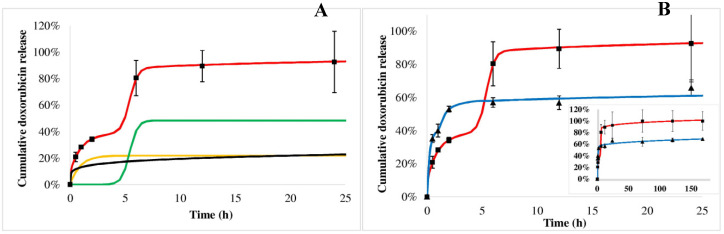
Doxorubicin cumulative release from PHB nanoparticles measured in acid (squares) and blood plasma (trigons). Colored lines show the modeled release profiles as follows: red line shows the release kinetics in the acid (**A**) built from the initial burst (yellow line), polymer degradation (green line) and diffusion (black line) in acid; blue line draws the release in blood plasma in (**B**). In the insert (**B**) the measured data and the fitted release profiles can be seen on the whole time scale.

The doxorubicin release from the PHB polymer can be characterized by complex kinetics. Figure 7A shows the measured values of the first 24 h (squares) in acid (similar to tumorous tissues) with the fitted triphasic curves (red line), where the yellow, green and black curves represent the initial burst, the polymer degradation and the slow diffusion, respectively. The modeled release profile (red line) can be drawn by combining these terms, which is harmonically fitted to the measured points (squares). Doxorubicin dissolution profiles are compared in acid (squares) and blood plasma (trigons) media (Figure 7B). Its release in plasma also follows the triphasic model (blue line), however, the initial burst is larger, while the second burst effect caused by the polymer degradation comes up substantially earlier, but to a smaller extent than that found in acid. The modeled release profile in blood plasma (blue line) also fits the measured values well. The initial burst of doxorubicin was 22% and 38%, while the *t_max_* values were 5.4 h and 1.7 h in acid and blood plasma, respectively.

**Figure 8:**
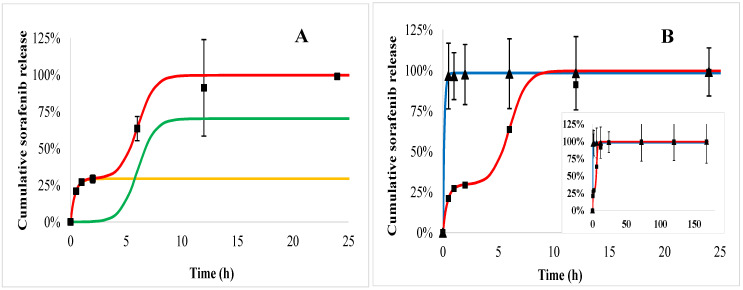
Sorafenib cumulative release from PHB nanoparticles measured in blood plasma (trigons) and acid (squares). The fitted release profiles in plasma (blue) and in acid (red) are also drawn. The modeled release profiles are shown as follows: the release kinetics in acid (**A**) was constructed from an initial burst (yellow line) and polymer degradation (green line). In the insert (**B**), the measured data and the fitted release profiles can be seen on the whole time scale.

Sorafenib dissolution from the PHB polymer in blood plasma (trigons) is so quick that the saturation occurs immediately after the initial burst (Figure 8B). In acid (Figure 8, squares) the initial burst is significantly lower (29%), followed by the polymer degradation phase, although before the third slow phase, all of the drug is released within 24 h.

**Figure 9:**
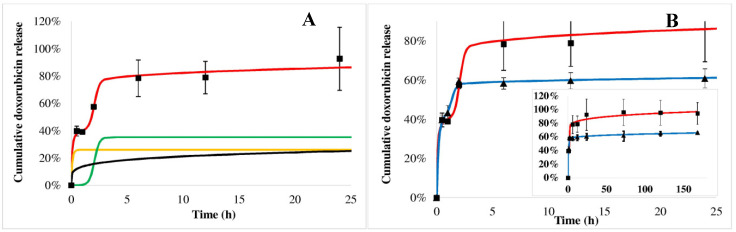
Doxorubicin cumulative release from PEGylated PHB nanoparticles measured in acid (squares) and blood plasma (trigons). Colored lines show the modeled release profiles as follows: red line draws the release kinetics in acid built from initial burst (yellow line), polymer degradation (green line) and diffusion (black line) in acid (**A**); blue line draws the release in blood plasma in the (**B**). In the insert (**B**), the measured data and the fitted release profiles can be seen on the whole time scale.

**Figure 10:**
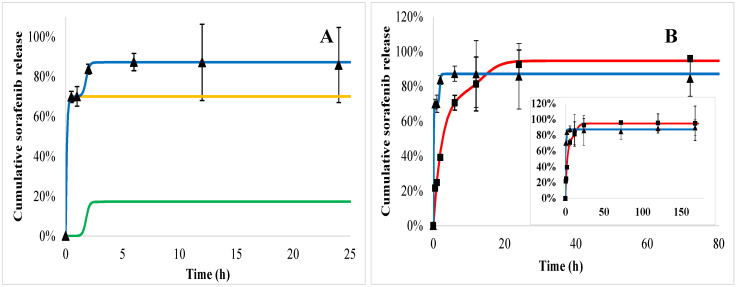
Sorafenib cumulative release from PEGylated PHB nanoparticles measured in blood plasma (trigons) and acid (squares). The fitted release profiles in plasma (blue) and in acid (red) are also drawn. The modeled release profiles are shown as follows: the release kinetics in acid (**A**) was built from the initial burst (yellow line) and polymer degradation (green line). In the insert (**B**), the measured data and the fitted release profiles can be seen on the whole time scale.

Doxorubicin release from the PEGylated PHB polymer (Figure 9) indicated also a triphasic profile similarly to PHB nanoparticles (Figure 7). t_max_ is smaller in plasma (Figure 9, trigons) than in acid (squares). In the case of sorafenib, a biphasic profile can be identified both in plasma and acid with an initial burst followed by the polymer degradation (Figure 10).

Comparing the release of active agents according to the polymers, it can be seen that the doxorubicin initial burst is slightly higher in both of the media from PEGylated PHB than from PHB, while the *t_max_* values are smaller for PEGylated PHB than for the PHB studied in the same medium (Table 2). This latter fact proves that the release exerted by the degradation of PEGylated PHB is quicker compared to that of PHB in both media. The diffusion exponent (‘n’) of Equation (3) can be calculated only in the case of doxorubicin release. It shows pH dependence with a significantly lower value in acid than in plasma, however, the type of polymer did not influence it substantially. When ‘n’ ≤ 0.43, the transport can be described with Fickian diffusion. [40] Similar correlation can be observed in the case of a diffusion kinetic constant (‘*k_d_*’). The drug dissolution derived from polymer degradation that is characterized by ‘*t_max_*’ was substantially smaller in blood plasma indicating a lower released fraction due to the degradation than under the acidic condition. The low value of ‘*t_max_*’ compared to that one generally published for PLGA can be explained by the low PHB molecular weight, which is expressed in the accelerated polymer degradation. [41]

In summary, the release of doxorubicin was sustained substantially in blood plasma. Its initial burst was 38% and 40% of the total loaded drug for PHB and PEGylated PHB nanoparticles, respectively, however it was slightly lower (22% and 26%) in the acidic media than in blood plasma. Until the end of the one-week study, 69% and 66% of the total entrapped doxorubicin were released from the dual drug-loaded PHB and PEGylated PHB nanotherapeutics, respectively, while these values were 100% and 94% in weak acid. The sorafenib release was substantially higher in acid than in blood plasma, which is advantageous considering the expected delivery in a tumorous environment. However, PHB nanoparticles with dual active agents released practically all of the sorafenib active agent initially, while PEG–PHB decreased its initial burst to 70% in blood plasma. Under acidic condition, the initial burst was from PHB (29%) and the PEG–PHB carriers (76%), while most of the sorafenib (99% and 93%, respectively) was released within 24 h in both cases. The strong affinity of sorafenib to plasma proteins explains its accelerated release in blood plasma, moreover, it can be concluded from the high initial burst, most of it must have been entrapped on the surface of the nanoparticles. [42]

In our nanotherapeutics for doxorubicin in blood plasma, after a general burst effect, the second and third phases were appropriately sustained, while in acidic conditions the large hydration of the polymer matrix and its quick dissolution took place.

Recently, we entrapped sorafenib and doxorubicin using PLGA and PEGylated PLGA nanocarriers [11], and experienced that the PEGylated PLGA showed faster drug release than PLGA, however, in that case, the PLGA was PEGylated by polymerization, and the ready PEGylated polymer was used for the dual drug microencapsulation. In contrast, with the PHB we applied PEG conjugation to the prepared sorafenib and doxorubicin co-loaded PHB nanoparticles. Comparing the results of the recent [11] and present study, we can conclude that the subsequently PEGylated PHB nanocarrier enabled slower release in blood plasma and accelerated dissolution in acidic pH for both sorafenib and doxorubicin related to the PLGA-based, which is beneficial for the co-administration of the drugs to tumorous tissues.

### 2.6. In Vitro Drug Release Kinetics

**Figure 11:**
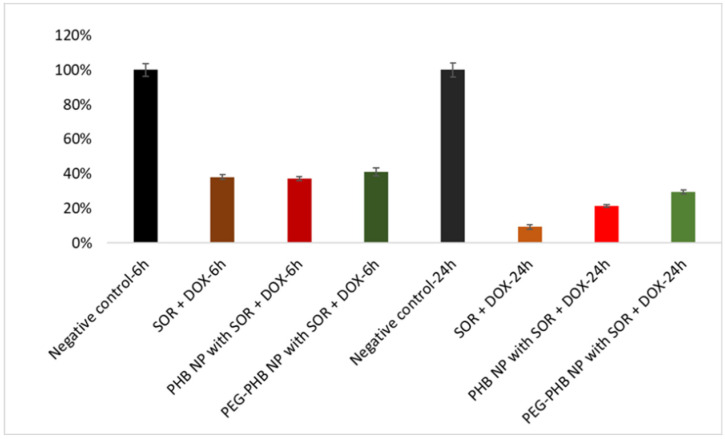
Viability of HCT-116 cells treated with sorafenib—(SOR)- and doxorubicin—(DOX)-loaded PHB and PEGylated-PHB nanoparticles and with the solution of drugs and that of untreated cells (negative control).

The cytotoxicity of the drug-loaded nanoparticles was investigated in HCT-116 colorectal carcinoma cells after 6 h and 24 h incubation. The viability of dual drug-loaded nanoparticles was compared to that of the drugs dissolved in dimethyl sulfoxide (DMSO), while the untreated cells were used as references (Figure 11). The non-toxic features of the PHB polymer have been proved previously [23]. The results showed that both of the PHB and the PEGylated-PHB nanoparticles including the two cytotoxic drugs performed similarly to the solution of the drugs after 6 h, while the cytotoxic effect of the nanoparticles was significantly increased after 24 h, however, it was slightly lower than that of the drugs solution.

## 3. Materials and Methods

### 3.1. Materials

AminoPEG *M_w_* = 5000 was purchased from Nanocs Inc. (New York, NY, USA), poly(vinyl alcohol) (PVA, *M_w_* = 30,000–70,000 g/mol, 87–90% hydrolysed), Pluronic F-68, dichloromethane (DCM), acetone, glacial acetic acid, dimethyl sulfoxide (DMSO), sodium azide, 1-ethyl-3(3-dimethylaminopropyl) carbodiimide (EDC), *N*-hydroxysuccinimide (NHS), sodium dodecyl sulphate (SDS), RPMI-1640 medium and triethyl amine (TEA) were obtained from Sigma Aldrich (Saint Louis, MO, USA). Sorafenib (free base) and doxorubicin hydrochloride were purchased from Active Biochem (Hong Kong, PRC). The following reagents were used for the [R,S]-*β*-butyrolactone oligomerization reaction: *β*-butyrolactone (BL, ≥98%) purified as described previously [43], tetrabutylammonium acetate (97%), Dowex^®^ 50WX8 (hydrogen form, 200–400 mesh) obtained from Sigma Aldrich (Saint Louis, MO, USA), as well as solvents: tetrahydrofuran over molecular sieves (THF, pure), *n*-hexane (pure p.a.), and chloroform (98.5%) obtained from Avantor Performance Materials Poland S.A. (Gliwice, Poland); all were used without further purification. The HCT-116 human colon carcinoma cell line was a kind gift from the National Institute of Oncology (Budapest, Hungary).

### 3.2. Synthesis of Encapsulating Polymers

In a moisture-free atmosphere (moisture < 1 ppm) at room temperature: the reactor equipped with a stirring bar was charged with tetrabutylammonium acetate (1.049 g, 3.48 mmol) and the initiator was dissolved in dry THF. Then, BL (5.11 mL, 5.4 g, 62.72 mmol) was added (initial concentration of 1 mol/L). The mixture was stirred vigorously at room temperature overnight. The final monomer conversion was determined by proton nuclear magnetic resonance (1H NMR) analysis based on the integral ratio of signals corresponding to methine (CH) protons of oligomer and monomer at δ = 5.24 ppm and δ = 4.77 ppm, respectively. The reaction was quenched with Dowex^®^ 50WX8 after completed monomer conversion. Then, the oligomer was precipitated into hexane after the cationite had been filtered off. Resulting material was dried under vacuum to a constant mass. SEC analysis of the poly([R,S]-3-hydroxybutyrate) revealed 2200 g/mol and ÐM = 1.3. Oligomers with Mw = 600 g/mol, Mw = 1300 g/mol and 1800 g/mol and molecular weight dispersity ÐM = 1.3, 1.6 and 1.3, respectively, (determined by gel permeation chromatography analysis) were prepared analogously.

### 3.3. Preparation of Nanoparticles by Nanoprecipitation

In preliminary experiments, the nanoprecipitation method was also investigated. Briefly, 15 mg PHB polymer was dissolved in 1.4 mL DCM. Then, 2 mg doxorubicin was dissolved in 0.2 mL acetone, and desalted with triethylamine (3× molar excess) overnight. In addition, 1 mg sorafenib and 1 mg doxorubicin free base dissolved in 0.1 mL acetone was added to the DCM solution. The 1.5 mL organic phase was added to the 15 mL aqueous solution containing 0.5% (m/m) PVA or Pluronic F-68 emulsifier. The organic solvent was evaporated by magnetic stirring overnight at room temperature and atmospheric pressure. The nanocomposite dispersions were centrifuged by a Hermle Z216 MK microcentrifuge (Gosheim, Germany) with 15,000 rpm for 20 min, washed and redispersed in an equal volume of MilliQ water three times.

### 3.4. Preparation of Nanoparticles by Emulsion Solvent Evaporation

The preparation of dual drug-loaded nanoparticles was a modified water-in-oil emulsion solvent evaporation method. Briefly, 10 mg doxorubicin hydrochloride was desalted with triethylamine in 1:3 molar ratio under 24 h stirring in 0.1 mL DMSO. The yield of the obtained free base was calculated assuming that the HCl content of the doxorubicin hydrochloride was completely removed from the drug in the process. The 20 mg PHB was dissolved in 1.0 mL DCM, then 0.05 mL DMSO solution with 0.5 mg doxorubicin free base and 1.5 mg sorafenib dissolved in 0.1 mL acetone were combined in a beaker and magnetically stirred for 30 s. The formed organic phase was added to the water phase which consisted of 6 mL MilliQ water with 0.5% (*w/v*) PVA. The emulsification was performed by sonication using Sonics Vibra Cell VCX 130 (Sonics & Materials Inc., Newtown, CT, USA) at an amplitude of 50% for 60 s. The organic solvents were evaporated by magnetic stirring for 4 h under atmospheric pressure at room temperature. Nanoparticles were centrifuged by a Hermle Z216 MK microcentrifuge (Gosheim, Germany) with 15,000 rpm for 20 min, washed thrice and redispersed in MilliQ water or phosphate buffered saline (PBS, pH 7.4).

### 3.5. Investigation of Nanoparticles

#### 3.5.1. Morphology and Particle Size

The morphology of nanoparticles was monitored after centrifuging 0.1 mL nanoparticle dispersion and their redispersion in MilliQ water, centrifugation as above and then washed three times. The dispersion was dropped onto a grid and dried at room temperature. Then, the FEI Apreo S scanning electron microscope was used for nanoparticle imaging.

The size distribution of the nanoparticles was determined by Zetasizer Nano ZS (Malvern Instruments, Malvern, UK) operated upon photon correlation spectroscopy. The particles were characterized by their intensity mean diameter and dispersity index.

#### 3.5.2. Yield, Drug Loading and Encapsulation Efficiency

The yield of the produced nanoparticles was determined by gravimetric measurement after washing and drying to a constant mass of 2 mL of nanoparticle suspension. The drug loading and encapsulation efficiency were investigated directly by dissolving 10 mg nanoparticles in 1 mL DMSO, and the solution was diluted to be detectable in the linear calibration range (1–50 µg/mL). For doxorubicin, the absorbance of the solutions was measured by T80 spectrophotometer (PG Instruments, Leicestershire, UK) at the absorbance maximum (480 nm). Sorafenib concentration was examined by high performance liquid chromatography (HPLC) at 270 nm in DMSO. The encapsulation efficiency of the active agents was calculated as follows:

Encaps. efficiency (%) = (mass of drug in nanocomposite/mass of total drug) × 100(1)

#### 3.5.3. FTIR Analysis

FTIR measurements were recorded on a Jasco FT/IR-4600 (Tokyo, Japan) system, equipped with a Jasco ATR Pro One single reflection diamond ATR accessory (incident angle 45°), and a DLATGS detector operating in the 4000–400 cm^−1^ interval. A resolution of 4 cm^−1^ and the co-addition of 64 individual spectra were applied. Prior to the evaluation, an ATR correction (Jasco Spectra Manager version 2, Spectra analysis module version 2.15.11, Tokyo, Japan) was performed on the raw spectra.

#### 3.5.4. TG–DSC–MSEGA Analysis

Thermal measurements were carried out on a Setaram LabsysEvo (Lyon, France) TG–DSC system, in a flowing (80 mL/min) high-purity (99.9999%) helium atmosphere in order to investigate the potential solvent residue in the PHB–sorafenib–doxorubicin nanoparticles. Dried samples were weighed directly into 100 μL Al crucibles (the reference cell was empty) and were heated from 20 °C to 190 °C with a heating rate of 20 °C/min. The obtained data were baseline corrected and further processed with the thermoanalyzer’s processing software (Calisto Processing, ver. 2.06, Setaram, Lyon, France). The thermal analyzer (both the temperature scale and calorimetric sensitivity) was calibrated by a multipoint calibration method, in which seven different certified reference materials were used to cover the thermal analyzer’s entire operating temperature range. In parallel with the thermal measurements, the analyses of evolved gases/volatiles were performed on a Omni Star™ mass spectrometric evolved gas analysis system (MSEGA, Pfeiffer Vacuum Gmbh, Asslar, Germany), which was connected to the aforementioned thermal analyzer. The gas splitter and transfer line to the mass spectrometer was thermostated to 200 °C. The measurement was done in SEM Bargraph Cycles acquisition mode, where the m/z interval of 11–110 was continuously scanned with a speed of 20 ms/amu. The spectrometer was operated in electron impact mode.

#### 3.5.5. PEGylation of Nanoparticles

The nanoparticle suspension of 10 mg/mL was centrifuged and washed with 1 mL MilliQ water and redispersed in 1 mL MilliQ water. The suspension was mixed with 0.2 mL aqueous solution of 50× molar excess of EDC and NHS related to the PHB concentration, then incubated for 60 min at 25 °C, and centrifuged and washed with MilliQ water. After the final centrifugation, the obtained carbodiimide-activated nanoparticles were redispersed in 2 mL PBS (pH 7.4) solution containing a 20× molar excess of aminoPEG and incubated for 1 h at 25 °C. Then, the nanocomposite dispersion was centrifuged, washed three times, and redispersed in 1 mL PBS.

#### 3.5.6. In Vitro Drug Release Study

The in vitro drug release of the PEGylated and non-PEGylated nanoparticles was investigated in human blood plasma (pH 7.4) and in ammonium acetate buffer (pH 5.5) which was used to represent the tumor microenvironment. For the in vitro release experiments, the original suspensions were centrifuged and washed 2 times, and an aliquot containing 5 mg nanoparticles was added to each 5 mL release media containing 0.03% sodium azide as bactericide in 5 mL non-transparent Eppendorf tubes, incubated at 37 °C in a G24 Environmental Incubator Shaker (New Brunswick Scientific Co. Inc., Edison, NJ, USA), and shaken by a BIO RS-24 Mini-rotator (Biosan, Riga, Latvia) for 7 days at 700 rpm. Three parallel samples per nanocomposite were incubated. Sampling was carried out after 0.5, 1, 2, 4, 6, 12, 24, 72, 120 and 168 h by taking 0.5 mL from each sample, centrifuging (Hermle Z216 MK microcentrifuge, Gosheim, Germany) for 20 min at 15,000 rpm, washing three times, and the pellet was dissolved in 0.5 mL DMSO.

The non-released sorafenib and doxorubicin concentration was measured by a Young Lin YL 9100 HPLC instrument with UV–Vis detection (YL Instruments Co. Ltd., Gyeonggi-do, Korea) at 30 °C. The active agents were separated by a Kinetex C18 column (150 mm × 4.6 mm, 5 µm; Phenomenex, Torrance, CA, USA). The mobile phase (0.1% tetrafluoroacetic acid in H_2_O: methanol) gradient is given in Table 3. The flow rate was adjusted to 1 mL/min. The detection wavelength of sorafenib and doxorubicin was 270 nm and 500 nm, respectively.

#### 3.5.7. Release Kinetics

Mathematical modeling of triphasic kinetics has been provided by Lucero-Acuña et al. [44,45]. The burst and relaxation induced and diffusion-controlled release is expressed in the first, second and third term of the Equation (1), respectively:

(2) $$\frac{M_{t}}{M_{\infty }}=\theta_{b}\left[1-exp\left(-k_{b}t\right)\right]+\theta_{r}\left\{\frac{exp\left[k_{r}\left(t-t_{max}\right)\right]}{1+exp\left[k_{r}\left(t-t_{max}\right)\right]}\right\}+\theta_{d}\left[1-\frac{6}{\pi^{2}}\overset{\infty }{\underset{n=1}{\sum }}\frac{1}{n^{2}}exp\left(-\frac{\pi^{2}n^{2}D_{c}t}{r_{1}^{2}}\right)\right]$$

Korsmeyer et al. (1983) [36] described the following semi-empirical equation:

Qd = ktn(3)

where ‘k’ is the diffusion kinetic rate constant that is determined by the geometric and structural properties of the drug-release system and ‘n’ is the diffusion exponent. By substituting the diffusion term in Equation (1) with this simplified Equation (2), Equation (1) can be transferred to:

(4) $$\frac{M_{t}}{M_{\infty }}=\theta_{b}\left[1-exp\left(-k_{b}t\right)\right]+\theta_{r}\left\{\frac{exp\left[k_{r}\left(t-t_{max}\right)\right]}{1+exp\left[k_{r}\left(t-t_{max}\right)\right]}\right\}+k_{d}t^{n}$$

where ‘*k_b_*’ is the initial burst constant, ‘*θ_b_*’ is the contribution of initial burst release over total mass drug release, ‘*k_r_*’ is the rate of degradation–relaxation constant, ‘*k_d_*’ is the diffusion kinetic constant and ‘*t_max_*’ is the time to achieve a maximum rate of drug release or the time to achieve 50% of release from the polymer degradation.

#### 2.5.8. Cytotoxicity Assay

The cytotoxicity of sorafenib- and doxorubicin-loaded PHB and PEGylated-PHB nanoparticles was investigated. Viability of HCT-116 colorectal carcinoma tumor cells was measured with a modified 3-(4,5-dimethylthiazol-2-yl)-2,5-diphenyltetrazolium bromide (MTT) assay based on ISO 19007:2018 standard method for investigating nanotoxicity. Briefly, the cells were seeded (10000 cells/well) in a 96-well plate and the nanoparticles with 5.0 μg sorafenib (SOR) and 1.55 μg doxorubicin (DOX) and 1.68 μg DOX, in PHB and PEGylated PHB, respectively, were added to 0.2 mL RPMI-1640 medium containing 10% FCS in another 96-well plate. In addition, 5 μg sorafenib and 1.55 μg doxorubicin were used as the control. Eight parallel wells were studied in each case. After 24 h, the pre-incubation of the cells and the release time of drug agents, the nanoparticles in the RPMI-1640 media were centrifuged and the cell media was changed for the nanoparticle-supernatant. This step is necessary to avoid nanoparticle sedimentation on tumor cells [46]. After 6/24 h incubation, the media were changed to 0.2 mL fresh, serum-free RPMI-1640 and 20 μL/well MTT reagent solution (5 mg MTT/mL) were added. After 2 h of incubation, the supernatant was removed, and 0.2 mL lysis solution (DMSO, 1% acetic acid, 10% SDS) was pipetted into each well. The cells containing the formed formazan crystals were dissolved during 5 min and with low shaking and the absorbance was measured at 570 nm by a plate reader (Thermo Scientific Multiscan Sky, Bio-science Kft., Budapest, Hungary). The viability of treated cells was related to the untreated cells (negative control).

## 4. Conclusions

PHBs were synthesized and tested for the co-encapsulation of sorafenib and doxorubicin anticancer agents. An emulsion–solvent evaporation method was developed for the efficient entrapment and co-delivery of the agents. Sorafenib and doxorubicin co-loaded PHB nanomedicines with desirable size, low dispersity and high encapsulation efficiency for both drugs were prepared. Thermal analysis indicated no residual solvent in the nanoparticle product. Substantially sustained drug release for doxorubicin was observed in blood plasma, however, the sorafenib release was accelerated. The release of drugs was quicker in acidic medium mimicking the tumor microenvironment. Subsequent PEGylation significantly improved the drug release profiles, while the physical and chemical characteristics of the nanomedicine remained appropriate. The PEGylated PHB represented more desirable release properties than our recently developed PLGA-based nanocarriers containing the same drugs. The time-dependent cytotoxic effect of the PHB and PEGylated PHB was observed, which was comparable to that of the drugs solution.

## Figures and Tables

**Table 1:** Yield, encapsulation efficiency (EE), doxorubicin (DOX) and sorafenib (SOR) loading of dual drug-loaded poly(3-hydroxybutyrate) (PHB) and PEGylated poly(3-hydroxybutyrate) (PEGylated PHB) nanoparticles.

	PHB	PEGylated PHB
Yield (% *w*/*w*)	66.9 ± 1.3	55.4 ± 1.4
EE (DOX) (%, *w*/*w*)	77 ± 3.7	64 ± 4.1
EE (SOR) (%, *w*/*w*)	84 ± 2.5	70 ± 1.2
DOX loading (%, *w*/*w*)	2.6	2.6
SOR loading (%, *w*/*w*)	8.4	7.7

**Table 2:** Kinetic parameters of the drug release mechanisms, where ‘n’ is the diffusion exponent, ‘*k_b_*’ is the initial burst constant, ‘*θ_b_*’ is the contribution of initial burst release over total mass drug release, ‘*k_r_*’ is the rate of the degradation–relaxation constant, ‘*k_d_*’ is the diffusion kinetic constant and ‘*t_max_*’ is the time to achieve a maximum rate of drug release derived from polymer degradation.

	*Ѳ_b_*	*k_b_* (1/h)	*Ѳ_r_*	*k_r_* (1/h)	*t_max_* (h)	*k_d_*	n
DOX_PHB in acid	0.22	1.1	0.48	2.8	5.4	0.132	0.17
DOX_PHB in plasma	0.38	4.36	0.16	4.5	1.7	0.022	0.38
DOX_PEG_PHB in acid	0.26	9.6	0.35	4.4	2.1	0.137	0.19
DOX PEG_PHB in plasma	0.40	5.5	0.16	>5.0	1.4	0.019	0.33
SOR_PHB in acid	0.29	2.5	0.70	>1.3	6.0	-	-
SOR_PHB in plasma	0.98	7.9	-	-	-	-	-
SOR_PEG_PHB in acid	0.76	0.4	0.18	0.4	14.0	-	-
SOR_PEG_PHB in plasma	0.70	>10.0	0.17	6.8	1.8	-	-

**Table 3:** The mobile phase (0.1% tetrafluoroacetic acid in H_2_O: methanol) gradient.

Time (min)	Tetrafluoroacetic Acid in H_2_O (%)	Methanol (%)
0.0	70.0	30.0
1.0	70.0	30.0
5.00	0.0	100.0
6.00	0.0	100.0
10.00	70.0	30.0
2.00	70.0	30.0
